# Development and internal validation of a lymphoma-specific nomogram for predicting venous thromboembolism: a retrospective cohort of 790 patients

**DOI:** 10.1186/s12885-025-15162-0

**Published:** 2025-11-05

**Authors:** Lili Pan, Wenzheng Lin, Yanyan Qiu, Jinhua Chen, Nainong Li, Tingbo Liu

**Affiliations:** 1 Department of Pediatric Hematology, Fujian Institute of Hematology, Fujian Provincial Key Laboratory on Hematology, Fujian Medical University Union Hospital, Fuzhou, China; 2 Department of Hematology, Fujian Institute of Hematology, Fujian Provincial Key Laboratory on Hematology, Fujian Medical University Union Hospital, Fuzhou, China; 3 Follow-up Center, Fujian Medical University Union Hospital, Fuzhou, China

**Keywords:** Thromboembolism, Lymphoma, Risk prediction, Nomogram

## Abstract

**Background:**

Thromboembolism (TE) is a serious complication in lymphoma, driving excess morbidity and mortality. Existing prediction tools perform suboptimally in lymphoma-specific settings.

**Methods:**

We retrospectively analysed 790 newly diagnosed lymphoma patients (January 2019–December 2021). Patients were randomly split 7:3 into development and internal-validation cohorts. Forty-eight candidate predictors were screened with LASSO, followed by multivariable Cox modelling to construct a nomogram. Discrimination and calibration were assessed at 6, 12 and 24 months using time-dependent ROC analysis and bootstrap calibration.

**Results:**

TE occurred in 77/790 patients (9.8%). Independent predictors were ECOG performance status, prior venous thromboembolism (VTE), coronary artery disease, central venous catheterisation, and APTT category. The nomogram showed good discrimination: AUCs were 0.813, 0.818 and 0.733 at 0.5, 1.0 and 2.0 years in the development cohort, and 0.724, 0.731 and 0.659 in the validation cohort. Conventional scores performed poorly in this population (e.g., at 1 year ThroLy 0.587 vs. Khorana 0.527). Calibration plots indicated close agreement between predicted and observed risks. Patients who experienced TE had poorer overall survival, with the greatest divergence in survival curves occurring within the first six months after diagnosis.

**Conclusions:**

This lymphoma-specific model improves TE risk stratification and can inform individualised prophylaxis and early monitoring. External, multi-centre validation is warranted to confirm generalisability.

**Supplementary Information:**

The online version contains supplementary material available at 10.1186/s12885-025-15162-0.

## Background

Thromboembolism is a serious complication in patients with cancer, occurring 4 to 7 times more frequently than in the general population, with a mortality risk that is 6 to 8 times higher [1–3]. Lymphoma patients are especially susceptible, with recent studies indicating that the incidence of TE in this group varies widely, from 4% to nearly 60%, depending on factors such as lymphoma subtype, disease stage, and the treatments they undergo [4–7]. As TE increases morbidity and early mortality, timely identification of high-risk patients is crucial for the prevention and management of TE events in lymphoma.

Although general cancer-associated TE risk scores, such as the Khorana score, have been widely used, their applicability to lymphoma is often limited [8]. In response, lymphoma-specific models, notably the ThroLy score, were developed and explicitly incorporate disease-related factors (e.g., raised lactate dehydrogenase and extranodal involvement) to provide more refined risk stratification [9]. External evaluations show that the ThroLy score—although demonstrating satisfactory predictive performance in several lymphoma cohorts—has exhibited variable discrimination across clinical settings, underscoring the need for refinement and real-world validation in larger, more heterogeneous populations [10, 11]. Likewise, the Khorana score has limited applicability in lymphoma because it does not capture disease-specific risk patterns [12] which further supports the development and use of lymphoma-specific tools to identify high-risk patients and guide thromboprophylaxis.

To facilitate bedside use, the present study pre-specified a simple, points-based clinical risk score derived from the final multivariable model; risk bands and absolute risks are reported to support prophylaxis decisions, with full details provided in the Results and discussed in terms of clinical applicability.

## Materials and methods

Data were collected from 790 newly diagnosed lymphoma patients treated at the Department of Hematology, Fujian Medical University Union Hospital, between January 2019 and December 2021. All cases were confirmed by pathological diagnosis according to the 2016 World Health Organization (WHO) classification. The full list of subtypes included in the study and their frequencies (counts and percentages) is provided in Supplementary Table S1. Exclusion criteria were: patients aged < 18 years; incomplete medical records; failure to complete the first chemotherapy cycle or discontinuation due to severe complications; and concurrent malignancies for which anti-tumour therapy was received within six months before or after the lymphoma diagnosis.

We analysed the clinical characteristics of newly diagnosed lymphoma patients, focusing on sex, age, body mass index (BMI), Eastern Cooperative Oncology Group (ECOG) performance status, history of venous thromboembolism, trauma or surgery within the preceding three months, and comorbidities including diabetes, hypertension, coronary artery disease and acute infection. We also recorded history of central venous catheterisation and smoking status. Baseline pre-treatment laboratory parameters included: red blood cell (RBC) count, haemoglobin (Hb), platelet (PLT) count, neutrophil count, white blood cell (WBC) count, prothrombin time (PT), international normalised ratio (INR), activated partial thromboplastin time (APTT), fibrinogen, thrombin time (TT), D-dimer, C-reactive protein (CRP), triglycerides (TG), high-density lipoprotein cholesterol (HDL-C), low-density lipoprotein cholesterol (LDL-C), apolipoprotein A, apolipoprotein B and lactate dehydrogenase (LDH).

Furthermore, we investigated associations between lymphoma-related factors and the incidence of thrombosis, including pathological subtype, Ann Arbor stage, use of granulocyte colony-stimulating factor (G-CSF), relapsed or refractory status, and the Khorana and ThroLy scores.

### Statistical analysis

Categorical variables were compared using the χ² test or Fisher’s exact test, as appropriate, whereas continuous variables were analysed with Student’s t test or the Mann–Whitney U test according to distribution. A multivariable Cox proportional hazards model incorporating variables with *P* < 0.10 in univariable screening was constructed to identify independent predictors of thromboembolism; the proportional hazards assumption was assessed using Schoenfeld residuals. For variables with < 10% missingness, data were imputed by multiple imputation using chained equations (m = 5). The initial set of 48 candidate predictors was reduced using LASSO regression prior to nomogram construction. Model performance was evaluated using time-dependent ROC curves and decision-curve analysis at 6, 12 and 24 months. All analyses were performed in R, version 4.3.0 (packages: rms, glmnet, timeROC, ggplot2). Two-sided *P* < 0.05 was considered statistically significant.

## Results

### Characteristics of the study population

**Fig. 1:**
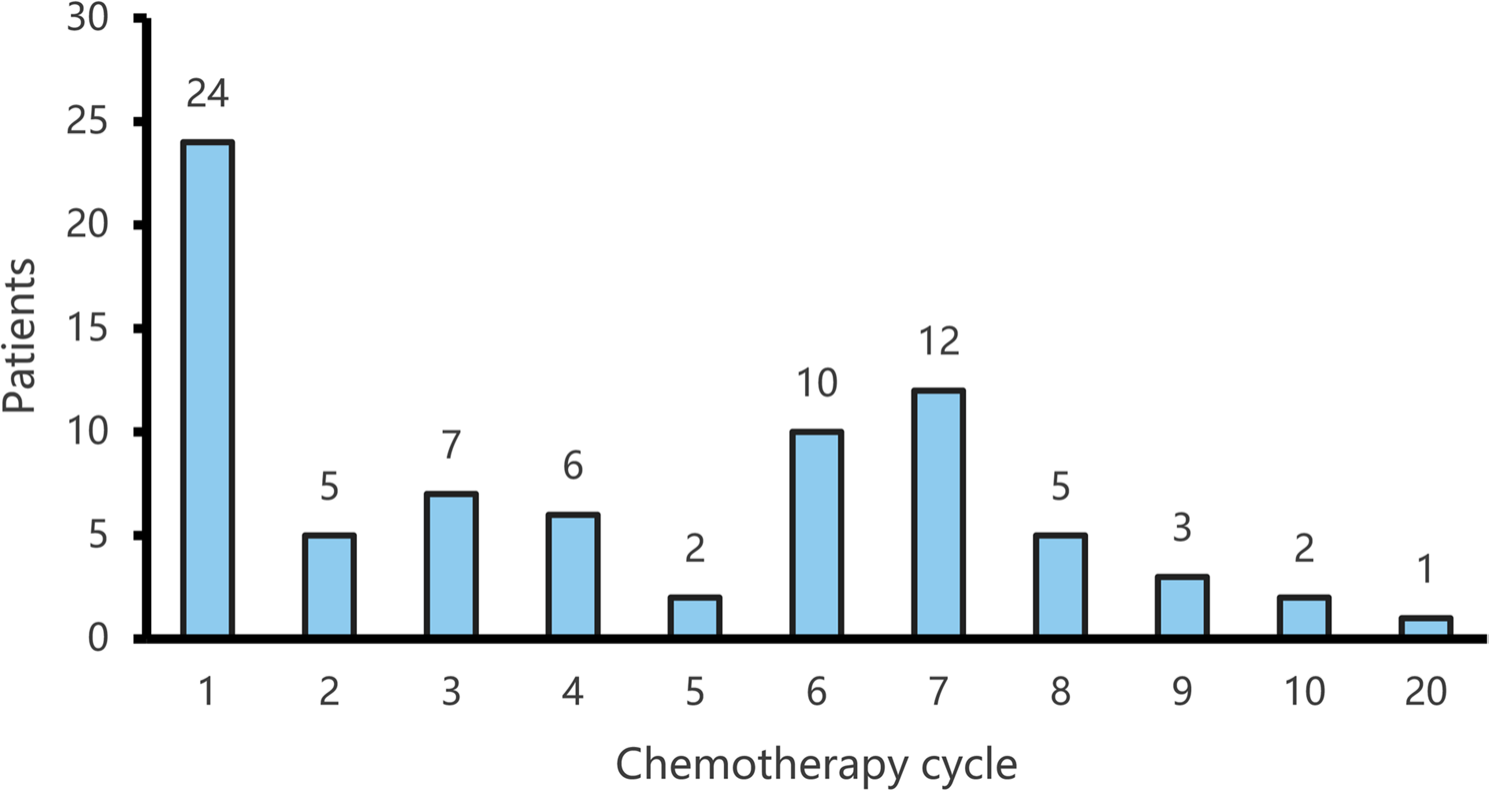
The completed chemotherapy cycle for lymphoma patients diagnosed with thrombosis

Among 790 patients with lymphoma, 77 developed thromboembolism (TE; 9.75%), with rates of 10.5% in non-Hodgkin lymphoma (NHL) and 4.6% in Hodgkin lymphoma (HL). The median time from the start of first-line chemotherapy (cycle 1, day 1) to TE diagnosis was 4 months. TE occurred most frequently after the first cycle (31.17%), followed by the seventh (15.58%), sixth (12.99%), and third (9.09%) cycles. Less frequent events occurred after the second, fourth and eighth cycles (each 6.49%), the fifth and tenth cycles (each 2.60%), and one case after 20 cycles (1.30%) (Fig. 1).

Of the 77 TE events, upper-limb venous thrombosis was most common (50/77, 64.9%), comprising deep-vein thrombosis (DVT; 49/77, 63.6%) and superficial-vein thrombosis (SVT; 1/77, 1.3%). Lower-limb DVT occurred in 14 patients (14/77, 18.2%), and intracranial venous thrombosis was observed in 10 patients (10/77, 13.0%). Internal jugular vein thrombosis was seen in 2 patients (2/77, 2.60%). Arterial thrombosis was uncommon (2/77, 2.6%; one lower-limb arterial event and one pulmonary arterial event). Anatomical categories were not mutually exclusive: seven patients had concurrent upper-limb DVT and SVT; one patient had DVT in both upper and lower limbs; and one patient had concomitant pulmonary arterial and lower-limb DVT. The single SVT-only case was a PICC-related upper-limb insertion-site vein event.

Regarding lymphoma subtypes, 45 patients (58.44%) had high-grade B-cell non-Hodgkin lymphoma, 17 (22.08%) had low-grade B-cell NHL, 3 (3.90%) had Hodgkin lymphoma, 2 (2.60%) had chronic lymphocytic leukaemia/small lymphocytic lymphoma (CLL/SLL), and 10 (12.99%) had other lymphomas, including T-cell and NK/T-cell types.

**Fig. 2:**
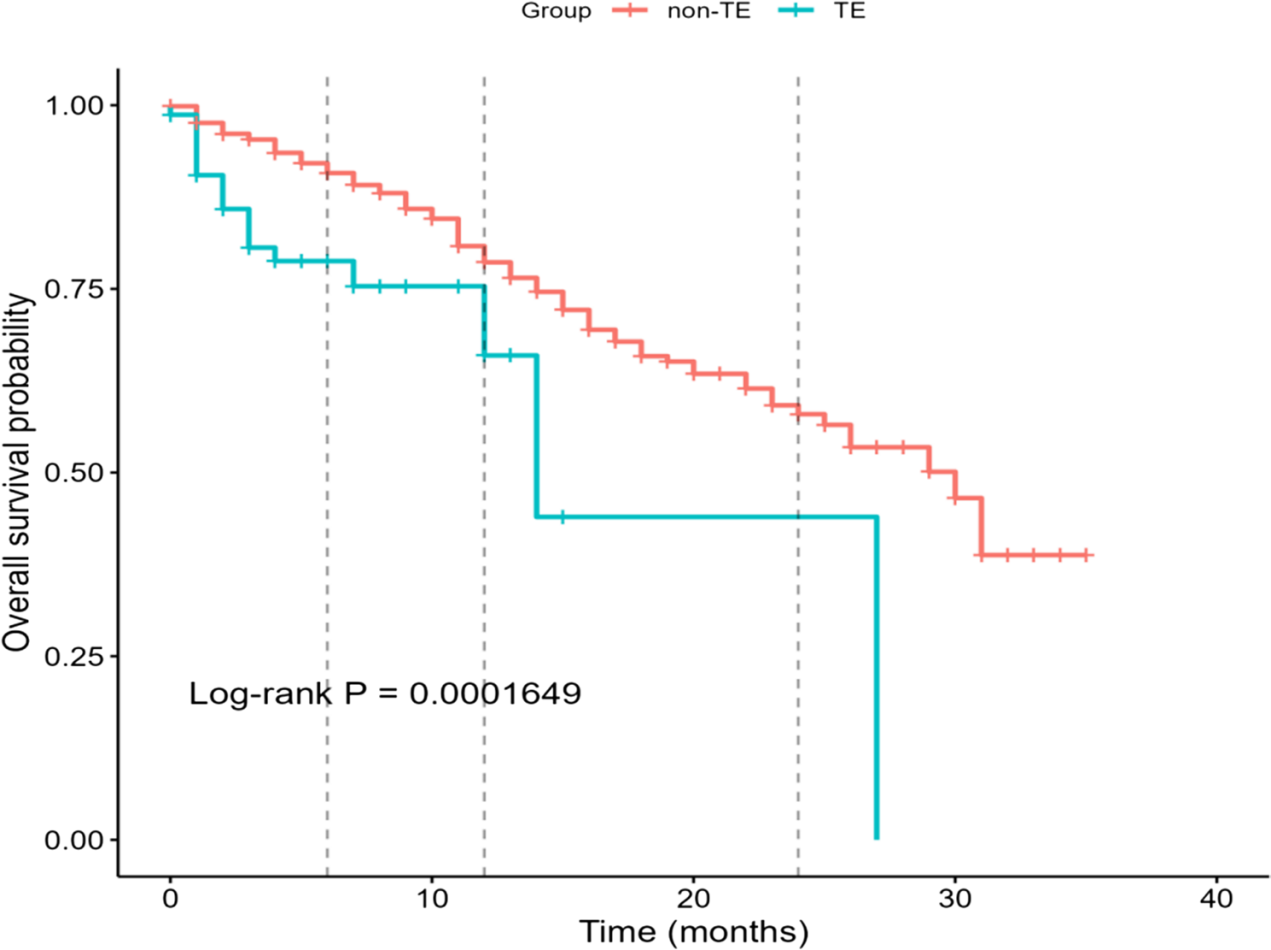
Kaplan–Meier overall survival curves: thromboembolism (TE) group vs. no-TE group

Over 35 months of follow-up, the Kaplan–Meier curves for overall survival differed significantly between groups (log-rank *P* = 0.000165; Fig. 2). By KM pointwise estimation, 6-month survival was 90.8% in the non-TE group versus 78.8% in the TE group (absolute difference 12.0% points; 95% CI 1.72–22.25; *P* = 0.022). Differences at 12 months (78.6% vs. 65.9%; *P* = 0.223) and 24 months (58.0% vs. 44.0%; *P* = 0.474) were not statistically significant, consistent with attenuation of the early separation over time.

Among the 77 patients with TE, 5 (6.50%) died due to thrombotic events, and 13 (16.88%) died as a result of disease progression or relapse. In addition, 2 (2.60%) experienced lower-limb functional impairment attributable to thrombosis, and 1 (1.30%) patient with intracranial thrombosis exhibited cognitive dysfunction.

### Analysis of the risk factors for TE in lymphoma patients

Baseline demographics and clinical characteristics were comparable between groups (Table 1). There were no significant differences in sex, age, BMI, histological subtype, Ann Arbor stage, Khorana score or ThroLy score. By contrast, patients who developed TE more often had a higher ECOG performance status, a history of coronary artery disease, concurrent infection, use of G-CSF, central venous catheterisation, and relapsed/refractory disease.

**Table 1:** Clinical characteristics of lymphoma patients with or without VTE

Variables	Overall (*n* = 790)	No-VTE (*n* = 713)	VTE (*n* = 77)	*P*-value
Age(year, mean)	58+−13.23	58+−12.98	59+−11.12	0.194
Gender				0.466
female	349 (44.18)	318 (44.60)	31 (40.26)	
male	441 (55.82)	395 (55.40)	46 (59.74)	
BMI (kg/m2)				0.481
≤18	67 (8.48)	61 (8.56)	6 (7.79)	
>18 and<30	706 (89.37)	638 (89.48)	68 (88.31)	
≥ 30	17 (2.15)	14 (1.96)	3 (3.90)	
ECOG (points, %)				0.049
0 or 1	695 (87.97)	631 (88.50)	64 (83.12)	
2 or 3	86 (10.89)	76 (10.66)	10 (12.99)	
≥ 4	9 (1.14)	6 (0.84)	3 (3.90)	
Smoke				0.742
Yes	163 (20.63)	146 (20.48)	17 (22.08)	
No	627 (79.37)	567 (79.52)	60 (77.92)	
Glycuresis				0.358
Yes	70 (8.86)	61 (8.56)	9 (11.69)	
No	720 (91.14)	652 (91.44)	68 (88.31)	
Hypertension				0.172
Yes	131 (16.58)	114 (15.99)	17 (22.08)	
No	659 (83.42)	599 (84.01)	60 (77.92)	
Coronary disease				0.015
Yes	22 (2.78)	16 (2.24)	6 (7.79)	
No	768 (97.22)	697 (97.76)	71 (92.21)	
History of VTE				0.065
Yes	10 (1.27)	7 (0.98)	3 (3.90)	
No	780 (98.73)	706 (99.02)	74 (96.10)	
Concurrent infection				0.011
Yes	305 (38.61)	265 (37.17)	40 (51.95)	
No	485 (61.39)	448 (62.83)	37 (48.05)	
Central venous catheterisation				0.002
Yes	596 (75.44)	527 (73.91)	69 (89.61)	
No	194 (24.56)	186 (26.09)	8 (10.39)	
Histological type (%)				0.433
Hodgkin	64 (8.10%)	61	3 (3.9%)	
high-grade NHL	429 (54.30%)	384	45 (58.44%)	
low-grade NHL	230 (29.11%)	213	17 (22.08%)	
T cell/NK-Tcell	127(16.08%)	117	10 (12.99%)	
CLL/SLL	17 (2.15%)	15	2 (2.6%)	
Recurrent/refractory lymphoma				0.014
Yes	51 (6.46)	41 (5.75)	10 (12.99)	
No	739 (93.54)	672 (94.25)	67 (87.01)	
Ann Arbor Stage				0.252
I	50 (6.33)	47 (6.59)	3 (3.90)	
II-III	273 (34.56)	240 (33.66)	33 (42.86)	
IV	467 (59.11)	426 (59.75)	41 (53.25)	
Khorana Score				0.249
0	572 (72.41)	519 (72.79)	53 (68.83)	
1	187 (23.67)	164 (23.00)	23 (29.87)	
2	31 (3.92)	30 (4.21)	1 (1.30)	
Throly Score				0.921
0–2	573 (72.53)	516 (72.37)	57 (74.02)	
3–5	214 (27.08)	194 (27.21)	20 (25.97)	
≥ 6	3 (0.38)	3 (0.42)	0	
G-CSF				0.008
Yes	452 (57.22)	397 (55.68)	55 (71.43)	
No	338 (42.78)	316 (44.32)	22 (28.57)	

Laboratory indices (white blood cell count, red blood cell count, haemoglobin, blood lipids, PT, APTT, TT, fibrinogen and D-dimer) were compared after categorisation using χ² tests. Only APTT showed a significant difference in distribution (χ² *P* = 0.029): shortened APTT (< 28 s) was more frequent in the TE group than in the non-TE group (5.19% vs. 0.98%), whereas the proportion with prolonged APTT (> 42 s) was similar (16.88% vs. 16.55%) (Supplementary Table S2).

A further COX multivariable analysis was performed based on the significant factors identified in the chi-square test. These factors included ECOG performance status, history of prior VTE, coronary artery disease, acute infection, central venous catheterisation, APTT, and the administration of granulocyte colony-stimulating factor. In the Cox model, ECOG 4 (vs. lower categories), prior VTE, coronary heart disease, and central venous catheterisation were independently associated with increased thrombotic risk (Table 2). By contrast, prolonged APTT > 42 s showed an inverse association with TE (HR 0.21, 95% CI 0.07–0.67; *P* = 0.008).

**Table 2:** Logistic regression analysis of factors affecting thrombosis

Variables	HR	95% CI	*P*-value
ECOG ≥ 4	6.00	1.86 ~ 19.40	0.003
Coronary disease	3.50	1.51 ~ 8.11	0.004
G-CSF	1.38	0.82–2.32	0.232
History of VTE	6.58	2.01 ~ 21.57	0.002
Central venous catheterisation	2.41	1.15 ~ 5.02	0.019
Concurrent infection	1.57	0.99 ~ 2.47	0.054
APTT > 42s	0.21	0.07 ~ 0.67	0.008
Recurrent/refractory lymphoma	1.74	0.89 ~ 3.39	0.104

### Development of a risk model for venous thromboembolism in lymphoma patients with cancer

**Fig. 3:**
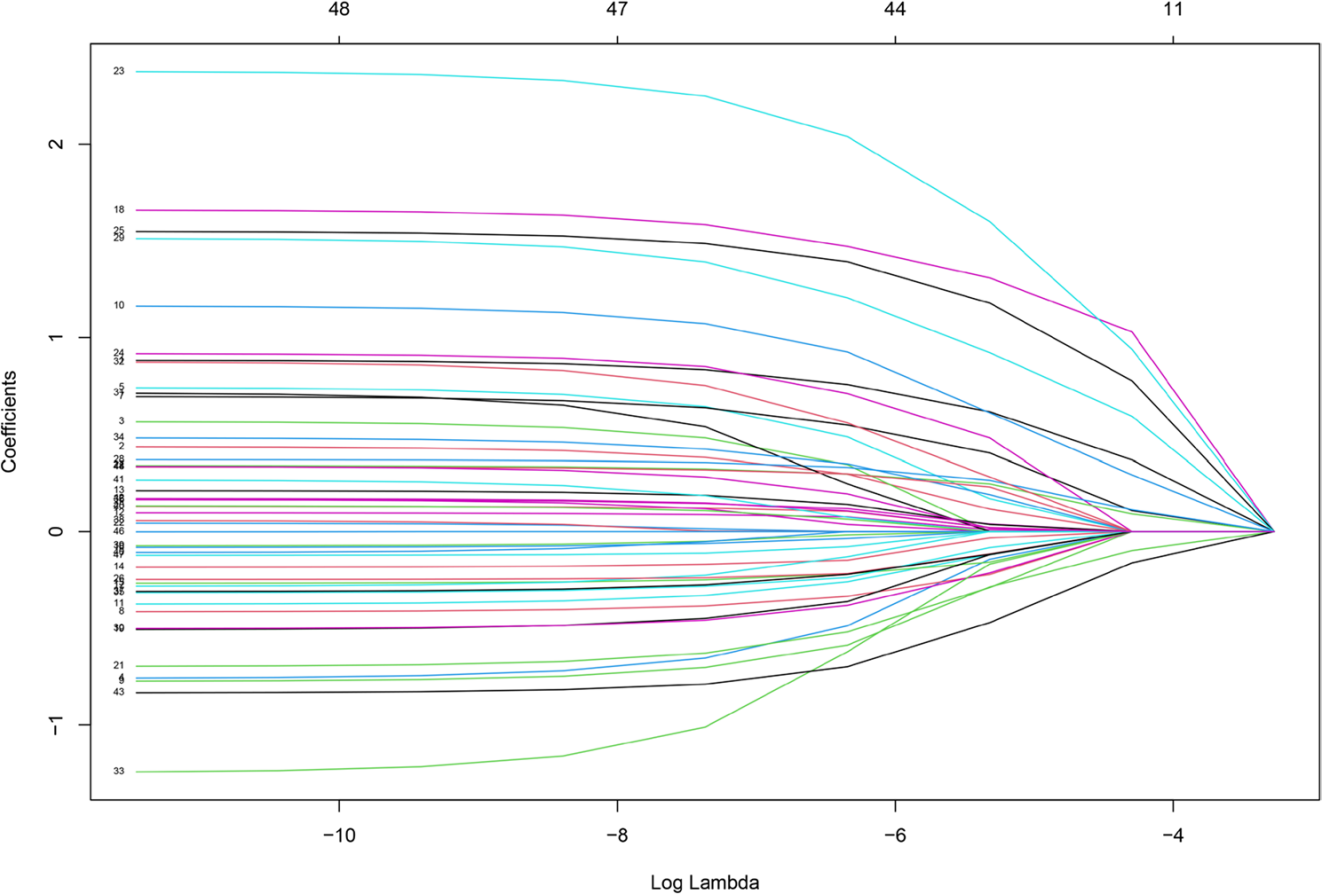
Lasso regression cross validation error chart

**Fig. 4:**
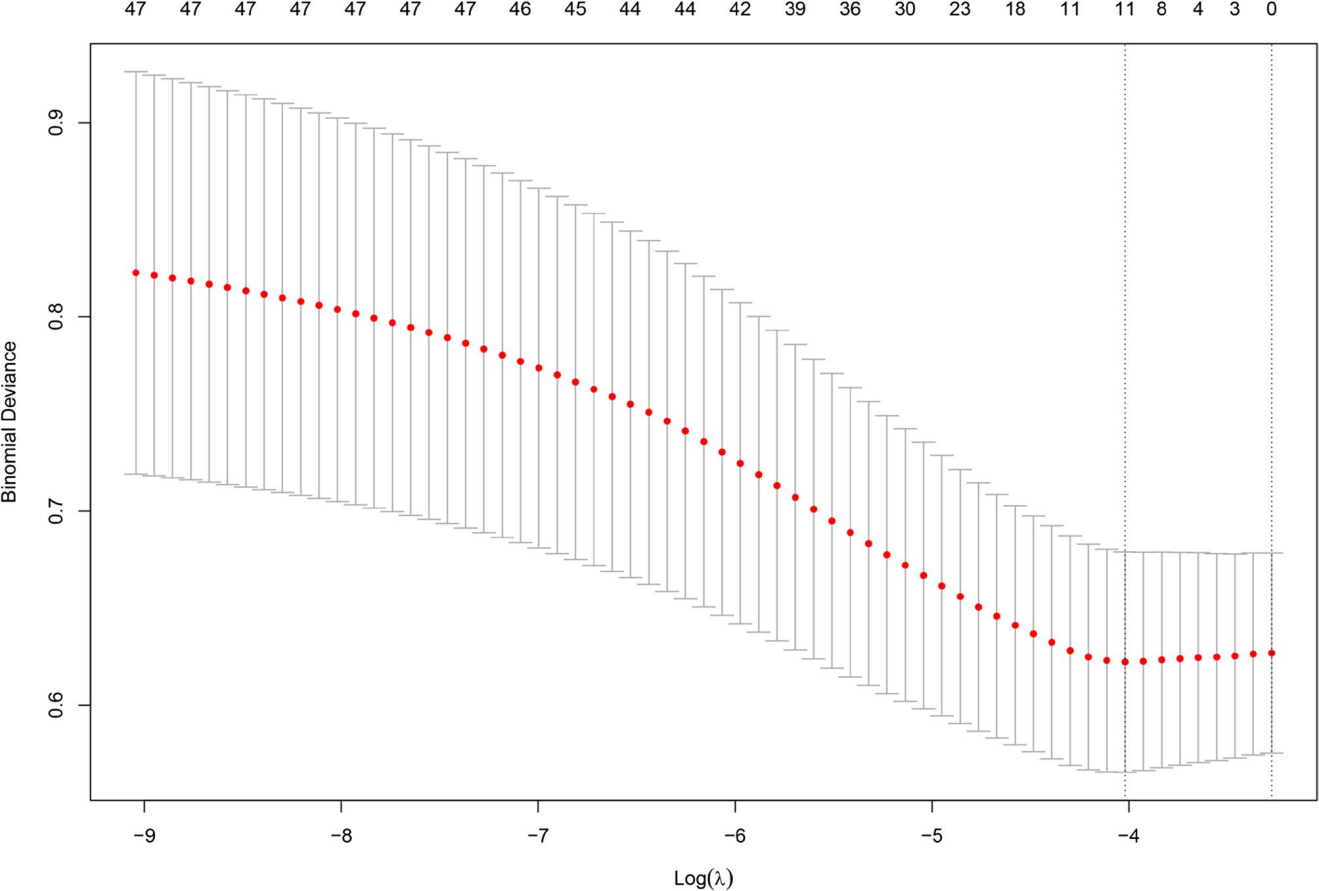
The variable selection process in Lasso regression

We developed a multivariable Cox model for venous thromboembolism and presented it as a nomogram to enable bedside estimation of absolute risk at prespecified horizons. The full cohort (*n* = 790) was randomly split 7:3 into a development set (*n* = 553) and an internal validation set (*n* = 237) using event-stratified sampling to preserve the case–mix and VTE incidence in both partitions. Forty-eight candidate predictors were considered a priori. After LASSO penalisation (Figs. 3 and 4), 11 variables were retained: ECOG performance status, serum creatinine, apolipoprotein A, apolipoprotein B, coronary heart disease, prior VTE, central venous catheterisation, acute infection, G-CSF use, relapsed/refractory status, and red cell count. Baseline distributions of these 11 predictors were comparable between the development and validation sets (all *P* > 0.05; Supplementary Table S3), supporting transportability of the model within this cohort.

In the development cohort, multivariable Cox regression of the 11 candidate predictors identified four independent risk factors for thrombosis in lymphoma: ECOG performance status (*P* = 0.019), central venous catheterisation (*P* = 0.023), prior venous thromboembolism (*P* = 0.003), and coronary heart disease (*P* = 0.004) (Supplementary Table S4).

**Fig. 5:**
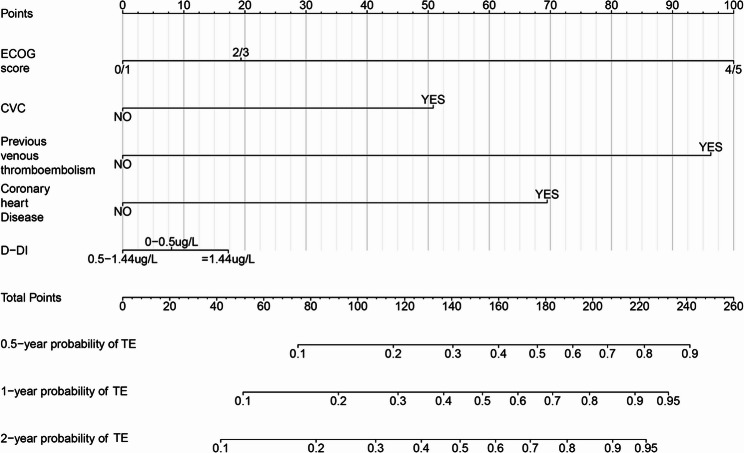
A nomogram model for predicting thrombus complications in lymphoma patients at 0.5, 1, and 2 years

Although D-dimer was not statistically significant in either univariable or multivariable analyses, it was retained in the prognostic model on clinical grounds, given its established relevance to thromboembolic risk and to maintain face validity of the nomogram. Based on the multivariable Cox model, we constructed a nomogram in the development cohort with thrombosis as the outcome and prespecified horizons at 0.5, 1.0 and 2.0 years (i.e. 6, 12 and 24 months) (Fig. 5). The lower panel of the nomogram displays the predicted probabilities of thromboembolic events at each time point; higher total points correspond to higher estimated risk.

**Fig. 6:**
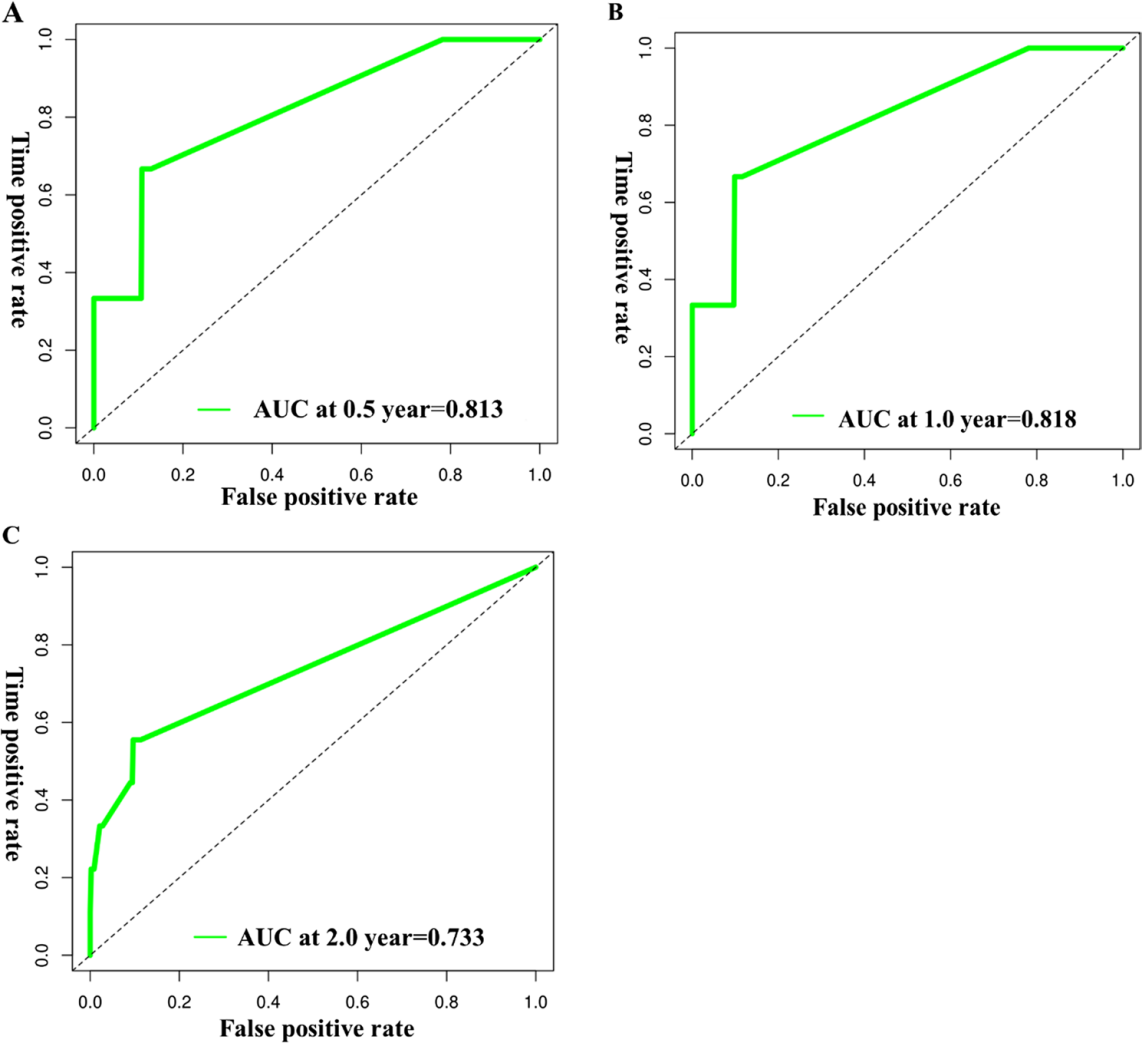
ROC curves for predicting the probability of concurrent TE at 0.5 years (**A**), 1 year (**B**) and 2 years (**C**) in patients with lymphoma in the model development group

**Fig. 7:**
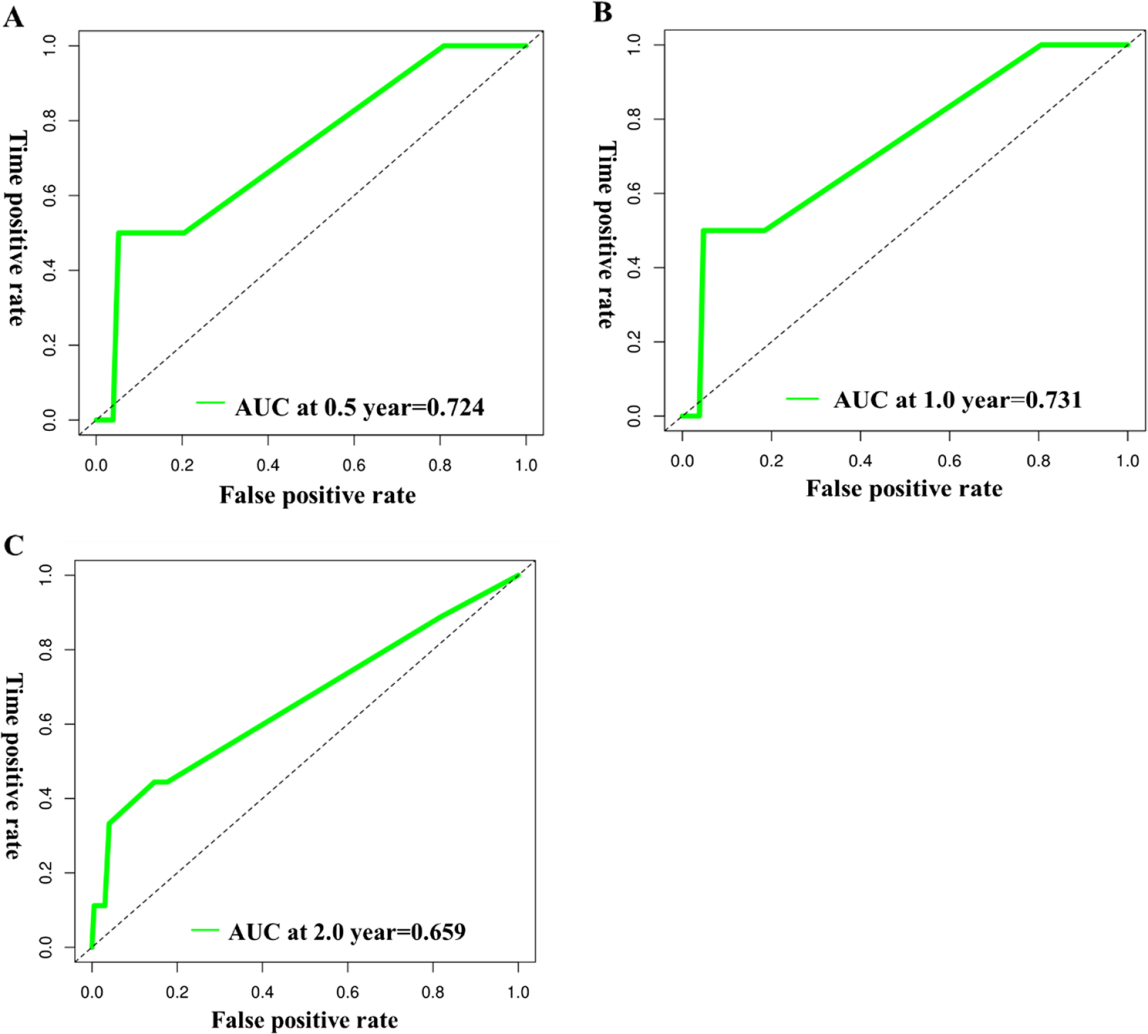
ROC curves for predicting the probability of 0.5 years (**A**), 1 year (**B**), and 2 years (**C**) concurrent TE in lymphoma patients in the validation

In the development cohort, time-dependent ROC curves at 0.5, 1.0 and 2.0 years yielded AUCs of 0.813, 0.818 and 0.733, respectively (mean across horizons 0.788), indicating good discrimination (Fig. 6). In the validation cohort, the corresponding AUCs were 0.724, 0.731 and 0.659 (mean 0.705), supporting acceptable out-of-sample performance (Fig. 7).

**Fig. 8:**
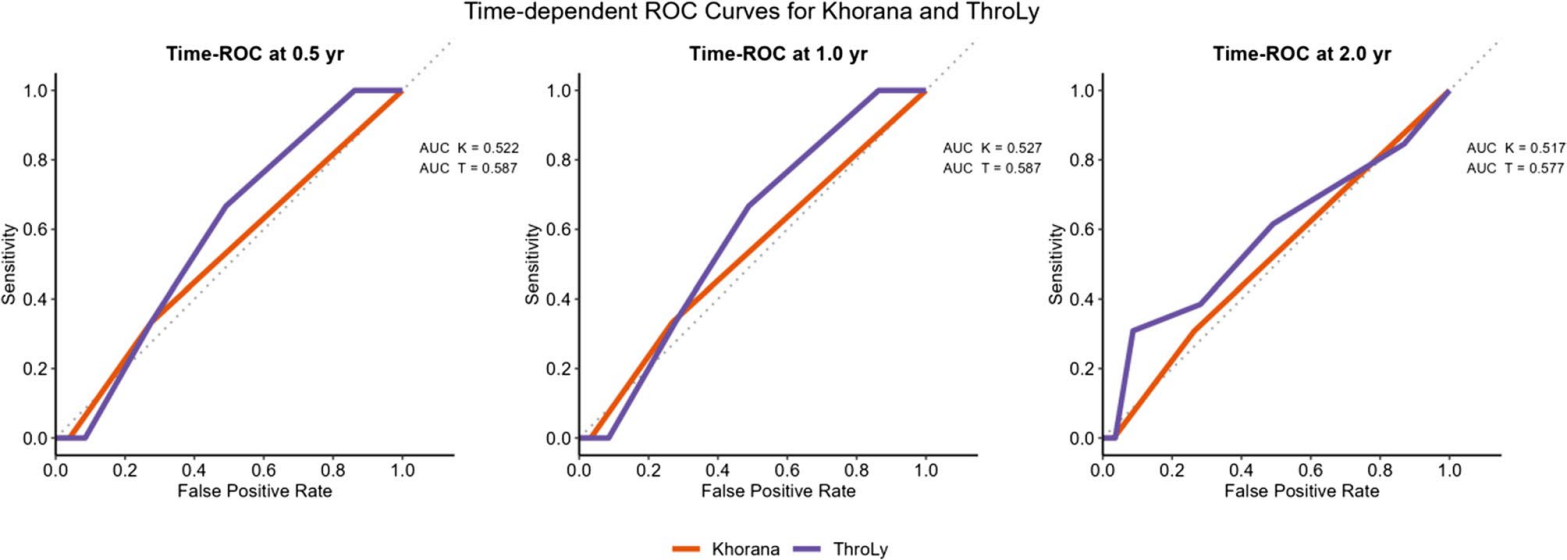
Time-dependent ROC curves comparing the Khorana and ThroLy scores in lymphoma patients. Panels show the discriminatory performance of the two conventional cancer-associated thrombosis (CAT) scores at 0.5 year (left), 1.0 year (centre) and 2.0 year (right) after diagnosis. The solid orange line represents the Khorana score; the solid purple line represents the ThroLy score

By comparison, the conventional scores showed limited discriminatory ability in lymphoma (Fig. 8). At 0.5 year, ThroLy achieved an AUC of 0.587, modestly higher than Khorana (0.522); similar patterns were seen at 1.0 year (0.587 vs. 0.527) and 2.0 years (0.577 vs. 0.517). Notably, all AUCs were < 0.60, underscoring the suboptimal performance of generic cancer-associated thrombosis scores in this population. Calibration plots (development and validation cohorts at 0.5, 1.0 and 2.0 years) showed generally good agreement between predicted and observed risks, with mild overprediction at 2 years, likely reflecting sparse late events.

## Discussion

Our study shows that thromboembolism is a frequent and clinically meaningful complication in lymphoma, particularly early in the treatment course. The derived lymphoma-specific model, which incorporates baseline clinical and laboratory variables, discriminated risk better than generic cancer scores: time-dependent AUCs were approximately 0.81 at 6–12 months, whereas both Khorana and ThroLy were consistently < 0.60 in our cohort. These findings support the use of disease-tailored tools for early and mid-term risk stratification rather than repurposing generic scores.

To facilitate bedside use, we provide a simple points-based score derived from the final multivariable model (Supplementary Table S5) together with a nomogram (Fig. 5). All predictors are available at baseline (before or at the start of first-line therapy): ECOG performance status, prior venous thromboembolism, coronary heart disease, central venous catheterisation (including PICC), and D-dimer. Integer points are summed to a total score, which maps to risk bands (low/intermediate/high); the corresponding absolute risks at 6, 12 and 24 months are read directly from the nomogram scales. The score is intended for use at baseline, before or at the initiation of systemic therapy, to identify patients who could benefit from targeted thromboprophylaxis, more cautious vascular-access choices and closer early monitoring. It is not a mandate for universal catheter avoidance; rather, it helps quantify trade-offs in patients with multiple risk factors.

The overall TE incidence was 9.75% (10.5% in non-Hodgkin lymphoma; 4.6% in Hodgkin lymphoma), aligning with previously published data [13, 14]. Arterial-only events were rare (one case); excluding this case would change the cohort incidence minimally (9.75% to 9.62%). Kaplan–Meier curves separated early and then converged: at 6 months, survival was lower in the TE group (absolute difference about 12% points), whereas differences at 12 and 24 months were not statistically significant. Consistent with recent literature that identifies TE as a leading cause of morbidity and mortality in lymphoma patients [10, 13, 15, 16]. Although TE was associated with worse overall survival and the largest separation in survival curves occurred within the first six months after diagnosis, this observation is descriptive and may be confounded by disease severity and other clinical factors (e.g., ECOG, relapsed disease). Early TE could plausibly contribute to adverse outcomes, but causality cannot be inferred from these observational data. Thus, the principal rationale for a lymphoma-specific score is to prevent early, morbid and occasionally fatal events, rather than to demonstrate a sustained long-term survival decrement.

Prior VTE and central venous catheterisation were strong baseline risk markers, underscoring the importance of device-related thrombosis in lymphoma care. Coronary heart disease was also associated with higher TE risk, likely reflecting clustering of pro-thrombotic comorbidities. With respect to coagulation markers, the between-group difference in APTT was driven by an excess of shortened APTT (< 28 s) among TE cases; prolonged APTT (>42 s) showed an inverse association in multivariable analysis. We did not perform systematic lupus-anticoagulant (LA) testing for all patients with prolonged APTT; therefore, prolonged APTT in our cohort likely reflects heterogeneous causes (e.g. LA, intrinsic factor deficiencies, anticoagulant exposure, or laboratory variability), which limits its specificity as an indicator of prothrombotic mechanisms. We retained D-dimer despite a non-significant independent effect because it is a clinically recognised indicator of coagulation activation and improves model face validity and bedside acceptance. The integration of D-dimer, despite its lack of statistical significance in multivariable analysis, further enhances the clinical relevance of our model. Although some studies have questioned the independent predictive value of D-dimer in cancer-associated thrombosis [17, 18], primarily due to the lack of well-defined cutoff values in cancer patients, particularly in hematologic malignancies, its established role as a marker of coagulation activation supports its inclusion in predictive models [19–23]. This decision aligns with the broader trend in recent studies that emphasize the importance of combining traditional risk factors with laboratory markers to improve the precision of thrombosis risk prediction [24–26].

Acute infection appeared to increase TE risk on univariable analysis but was not independently associated after adjustment.This remains biologically plausible—systemic inflammation, endothelial activation and immune-mediated thrombogenesis can amplify the baseline hypercoagulability seen in haematological malignancies [27–30]. Given the higher infection burden in lymphoma, prospective work that captures infection timing, severity and treatment is warranted. G-CSF has mechanistic links to thrombosis through neutrophilia and NET formation [31–34]. In our cohort, however, G-CSF use was not an independent predictor after adjustment; the predominant use of short-acting preparations may have attenuated any association. We therefore view G-CSF as a context variable rather than a stand-alone trigger for prophylaxis decisions, pending confirmatory studies.

This was a single-centre study with internal validation; event counts, particularly in the validation subset and at later horizons, limit precision. We focused on variables available at baseline to avoid look-ahead bias; post-baseline treatment response was not modelled. Calibration was primarily assessed visually; numerical metrics can be unstable with few events and heavy censoring but will be provided in larger, independent external validation. Death can preclude TE; our primary estimand was the cause-specific hazard for incident TE to inform baseline risk stratification, and we clarify this in the Methods.

## Supplementary Information

Supplementary Material 1.

Supplementary Material 2.

## Data Availability

The datasets supporting the conclusions of this article are not publicly available due to data protection for enrolled centers but are available from the corresponding author on reasonable request.

## Abbreviations

TE: Thromboembolism
VTE: Venous thrombo-embolism
ECOG: Eastern cooperative oncology group performance status
WHO: World health organization, BMI, body mass index
LDH: Lactate dehydrogenase
NHL: Non-hodgkin lymphoma
HL: Hodgkin lymphoma
CLL/SLL: Chronic lymphocytic leukaemia/small lymphocytic lymphoma
G-CSF: Granulocyte-colony stimulating factor
PT: Prothrombin time
INR: International normalised ratio
APTT: Activated partial thromboplastin time
TT: Thrombin time
FIB: Fibrinogen
D-DI: D-dimer
CRP: C-reactive protein
TG: Triglycerides
HDL-C: High-density lipoprotein cholesterol
LDL-C: Low-density lipoprotein cholesterol
RBC: Red blood cell count
Hb: Haemoglobin
PLT: Platelet count
WBC: White blood cell count
