# Supplementary material for: Development and internal validation of a lymphoma-specific nomogram for predicting venous thromboembolism: a retrospective cohort of 790 patients
Source: BMC Cancer. 2025 Nov 5;25:1720. doi: 10.1186/s12885-025-15162-0 (PMC12590811; doi:10.1186/s12885-025-15162-0)
Supplement: Supplementary file 2 — Supplementary Material 2. [file 12885_2025_15162_MOESM2_ESM.docx]

Supplement Table S1**.** Lymphoma subtypes and frequencies (WHO 2016 classification).

| **Lymphoma subtype** | **Cases** | **Proportion** |
| --- | --- | --- |
| Diffuse large B-cell lymphoma | 377 | 47.72% |
| Follicular lymphoma | 94 | 11.90% |
| Marginal zone lymphoma | 62 | 7.85% |
| Hodgkin lymphoma | 58 | 7.34% |
| Other lymphomas | 36 | 4.56% |
| Mantle cell lymphoma | 34 | 4.30% |
| Extranodal NK/T-cell lymphoma | 28 | 3.54% |
| Other T-cell lymphomas | 23 | 2.91% |
| Angioimmunoblastic T-cell lymphoma | 22 | 2.78% |
| Peripheral T-cell lymphoma, NOS | 18 | 2.28% |
| Burkitt lymphoma | 16 | 2.03% |
| Small B-cell lymphoma | 8 | 1.01% |
| T-lymphoblastic lymphoma/leukaemia | 8 | 1.01% |
| Anaplastic large cell lymphoma | 6 | 0.76% |

Supplement Table S2**.** Comparison of laboratory tests in lymphoma patients with and without thrombus formation.

| Variables | Overall(n=790) | No-VTE (n=713) | VTE (n=77) | P-value |
| --- | --- | --- | --- | --- |
| WBC (*10^9/L) |  |  |  | 0.660 |
| ＜4 | 139 (17.59) | 127 (17.81) | 12 (15.58) |  |
| 4-10 | 528 (66.84) | 473 (66.34) | 55 (71.43) |  |
| ＞10 | 123 (15.57) | 113 (15.85) | 10 (12.99) |  |
| N (*10^9/L) |  |  |  | 0.808 |
| ＜1.5 | 75 (9.49) | 69 (9.68) | 6 (7.79) |  |
| 1.5-7*10^9/L | 566 (71.65) | 511 (71.67) | 55 (71.43) |  |
| ＞7*10^9/L | 149 (18.86) | 133 (18.65) | 16 (20.78) |  |
| Hb (g/L) |  |  |  | 0.051 |
| ＜110 | 251 (31.77) | 223 (31.28) | 28 (36.36) |  |
| 110-150 | 497 (62.91) | 456 (63.96) | 41 (53.25) |  |
| ＞150 | 42 (5.32) | 34 (4.77) | 8 (10.39) |  |
| RBC (*10^12L) |  |  |  | 0.224 |
| ＜3.5 | 154 (19.49) | 134 (18.79) | 20 (25.97) |  |
| 3.5-5.5 | 619 (78.35) | 564 (79.10) | 55 (71.43) |  |
| ＞5.5 | 17 (2.15) | 15 (2.10) | 2 (2.60) |  |
| PLT (*10^9/L) |  |  |  | 0.562 |
| ＜100 | 88 (11.14) | 82 (11.50) | 6 (7.79) |  |
| 100-300 | 519 (65.7) | 468 (65.64) | 51 (66.23) |  |
| ＞300 | 183 (23.16) | 163 (22.86) | 20 (25.97) |  |
| CRP (mg/L) |  |  |  | 0.966 |
| 0-8 | 443 (56.08) | 400 (56.10) | 43 (55.84) |  |
| ＞8 | 347 (43.92) | 313 (43.90) | 34 (44.16) |  |
| PT (s) |  |  |  | 0.293 |
| ＜11 | 12 (1.52) | 11 (1.54) | 1 (1.30) |  |
| 11-15 | 741 (93.8) | 671 (94.11) | 70 (90.91) |  |
| ＞15 | 37 (4.68) | 31 (4.35) | 6 (7.79) |  |
| INR |  |  |  | 0.465 |
| ＜0.8 | 5 (0.63) | 4 (0.56) | 1 (1.30) |  |
| 0.8-1.5 | 763 (96.58) | 688 (96.49) | 75 (97.40) |  |
| ＞1.5 | 22 (2.78) | 21 (2.95) | 1 (1.30) |  |
| APTT (s) |  |  |  | 0.029 |
| ＜28 | 11 (1.39) | 7 (0.98) | 4 (5.19) |  |
| 28-42 | 648 (82.03) | 588 (82.47) | 60 (77.92) |  |
| ＞42 | 131 (16.58) | 118 (16.55) | 13 (16.88) |  |
| FIB(g/L） |  |  |  | 0.768 |
| ＜2g/L | 17 (2.15) | 15 (2.10) | 2 (2.60) |  |
| 2-4 g/L | 477 (60.38) | 429 (60.17) | 48 (62.34) |  |
| ＞4 g/L | 296 (37.47) | 269 (37.73) | 27 (35.06) |  |
| TT (s) |  |  |  | 0.450 |
| ＜14 | 20 (2.53) | 17 (2.38) | 3 (3.90) |  |
| 14-21 | 756 (95.7) | 684 (95.93) | 72 (93.51) |  |
| ＞21 | 14 (1.77) | 12 (1.68) | 2 (2.60) |  |
| DD (ug/ml) |  |  |  | 0.429 |
| 0.5-1.44 | 242 (30.63) | 220 (30.86) | 22 (28.57) |  |
| 0-0.5 | 321 (40.63) | 293 (41.09) | 28 (36.36) |  |
| ＞1.44 | 227 (28.73) | 200 (28.05) | 27 (35.06) |  |
| TG (mmol/L) |  |  |  | 0.963 |
| 0.4-1.86 | 607 (76.84) | 548 (76.86) | 59 (76.62) |  |
| ＜0.4 | 183 (23.16) | 165 (23.14) | 18 (23.38) |  |
| HDL-C (mmol/L) |  |  |  | 0.137 |
| ＜0.9 | 320 (40.51) | 287 (40.25) | 33 (42.86) |  |
| 0.9-1.9 | 452 (57.22) | 412 (57.78) | 40 (51.95) |  |
| ＞1.9 | 18 (2.28) | 14 (1.96) | 4 (5.19) |  |
| LDL (mmol/L) |  |  |  | 0.553 |
| ＜1.1 | 39 (4.94) | 34 (4.77) | 5 (6.49) |  |
| 1.1-3.5 | 577 (73.04) | 524 (73.49) | 53 (68.83) |  |
| ＞3.5 | 174 (22.03) | 155 (21.74) | 19 (24.68) |  |
| aPOA (g/L) |  |  |  | 0.418 |
| ＜1.0 | 227 (28.77) | 200 (28.09) | 27 (35.06) |  |
| 1.0-1.6 | 502 (63.62) | 458 (64.33) | 44 (57.14) |  |
| ＞1.6 | 60 (7.6) | 54 (7.58) | 6 (7.79) |  |
| aPOB (g/L) |  |  |  | 0.117 |
| ＜0.6 | 50 (6.34) | 45 (6.32) | 5 (6.49) |  |
| 0.6-1.1 | 529 (67.05) | 485 (68.12) | 44 (57.14) |  |
| ＞1.1 | 210 (26.62) | 182 (25.56) | 28 (36.36) |  |
| Serum creatinine |  |  |  | 0.149 |
| ＜41umol/L | 34 (4.3) | 34 (4.77) | 0 (0.00) |  |
| 41-73umol/L | 512 (64.81) | 461 (64.66) | 51 (66.23) |  |
| ＞73umol/L | 244 (30.89) | 218 (30.58) | 26 (33.77) |  |
| LDH |  |  |  | 0.808 |
| ＜245IU/L | 431 (54.65) | 390 (54.7) | 41 (53.25) |  |
| ＞245IU/L | 359 (45.44) | 323 (45.30) | 36 (46.75) |  |

PT, prothrombin time; APTT, activated partial thromboplastin time; TT, thrombin time; DD, D-dimer; FIB, plasma fibrinogen.

Supplement Table S3. Comparison of feature variables between model development group and validation group

| Variables, n (%) | Overall  (n = 790) | Model development group  (n = 553) | Validation group  (n = 237) | P-value |
| --- | --- | --- | --- | --- |
| ECOG (points, %) |  |  |  | 0.559 |
| 0 or 1 | 695 (87.97) | 491 (88.79) | 204 (86.08) |  |
| 2 or 3 | 86 (10.89) | 56 (10.13) | 30 (12.66) |  |
| ≥4 | 9 (1.14) | 6 (1.08) | 3 (1.27) |  |
| Serum creatinine |  |  |  | 0.122 |
| ＜41umol/L | 34 (4.3) | 22 (3.98) | 12 (5.06) |  |
| 41-73umol/L | 512 (64.81) | 371 (67.09) | 141 (59.49) |  |
| ＞73umol/L | 244 (30.89) | 160 (28.93) | 84 (35.44) |  |
| aPOA (g/L) |  |  |  | 0.158 |
| ＜1.0 | 227 (28.77) | 148 (26.76) | 79 (33.47) |  |
| 1.0-1.6 | 502 (63.62) | 361 (65.28) | 141 (59.75) |  |
| ＞1.6 | 60 (7.6) | 44 (7.96) | 16 (6.78) |  |
| aPOB (g/L) |  |  |  | 0.407 |
| ＜0.6 | 50 (6.34) | 38 (6.87) | 12 (5.08) |  |
| 0.6-1.1 | 529 (67.05) | 374 (67.63) | 155 (65.68) |  |
| ＞1.1 | 210 (26.62) | 141 (25.50) | 69 (29.24) |  |
| Coronary disease |  |  |  | 0.777 |
| Yes | 768 (97.22) | 537 (97.11) | 231 (97.47) |  |
| No | 22 (2.78) | 16 (2.89) | 6 (2.53) |  |
| History of VTE |  |  |  | 0.728 |
| Yes | 780 (98.73) | 545 (98.55) | 235 (99.16) |  |
| No | 10 (1.27) | 8 (1.45) | 2 (0.84) |  |
| Central venous catheterisation |  |  |  | 0.971 |
| Yes | 194 (24.56) | 136 (24.59) | 58 (24.47) |  |
| No | 596 (75.44) | 417 (75.41) | 179 (75.53) |  |
| G-CSF |  |  |  | 0.826 |
| Yes | 338 (42.78) | 238 (43.04) | 100 (42.19) |  |
| No | 452 (57.22) | 315 (56.96) | 137 (57.81) |  |
| Concurrent infection |  |  |  | 0.380 |
| Yes | 485 (61.39) | 345 (62.39) | 140 (59.07) |  |
| No | 305 (38.61) | 208 (37.61) | 97 (40.93) |  |
| Recurrent/refractory |  |  |  | 0.394 |
| Yes | 739 (93.54) | 520 (94.03) | 219 (92.41) |  |
| No | 51 (6.46) | 33 (5.97) | 18 (7.59) |  |
| RBC (*10^12L) |  |  |  | 0.739 |
| ＜3.5 | 154 (19.49) | 105 (18.99) | 49 (20.68) |  |
| 3.5-5.5 | 619 (78.35) | 435 (78.66) | 184 (77.64) |  |
| ＞5.5 | 17 (2.15) | 13 (2.35) | 4 (1.69) |  |

Supplement Table S4. Multivariate analysis of characteristic variables in lymphoma patients in the model development group

| Variables | HR | 95% CI | P-value |
| --- | --- | --- | --- |
| ECOG | 1.82 | 1.1-3.01 | 0.019 |
| Coronary disease | 3.40 | 1.47-7.86 | 0.004 |
| G-CSF | 1.27 | 0.71-2.24 | 0.420 |
| History of VTE | 5.97 | 1.87-19.05 | 0.003 |
| Central venous catheterisation | 2.35 | 1.12-4.9 | 0.023 |
| Concurrent infection | 1.44 | 0.89-2.32 | 0.135 |

Supplement Table S5. Points-based clinical score derived from the multivariable model. Sum the points across present categories to obtain a total score; classify into low/intermediate/high risk and read 6-, 12- and 24-month absolute risks from the nomogram (Figure 5).

| **Predictor** | **Category** | **Points** |
| --- | --- | --- |
| ECOG score | 0–1 | 0 |
|  | 2–3 | 20 |
|  | 4 | 100 |
| Central venous catheterisation (incl. PICC) | No | 0 |
|  | Yes | 50 |
| Prior venous thromboembolism | No | 0 |
|  | Yes | 95 |
| Coronary heart disease | No | 0 |
|  | Yes | 70 |
| D-dimer | 0–0.5 µg/L | 7.5 |
|  | 0.5–1.44 µg/L | 0 |
|  | ≥1.44 µg/L | 17.5 |


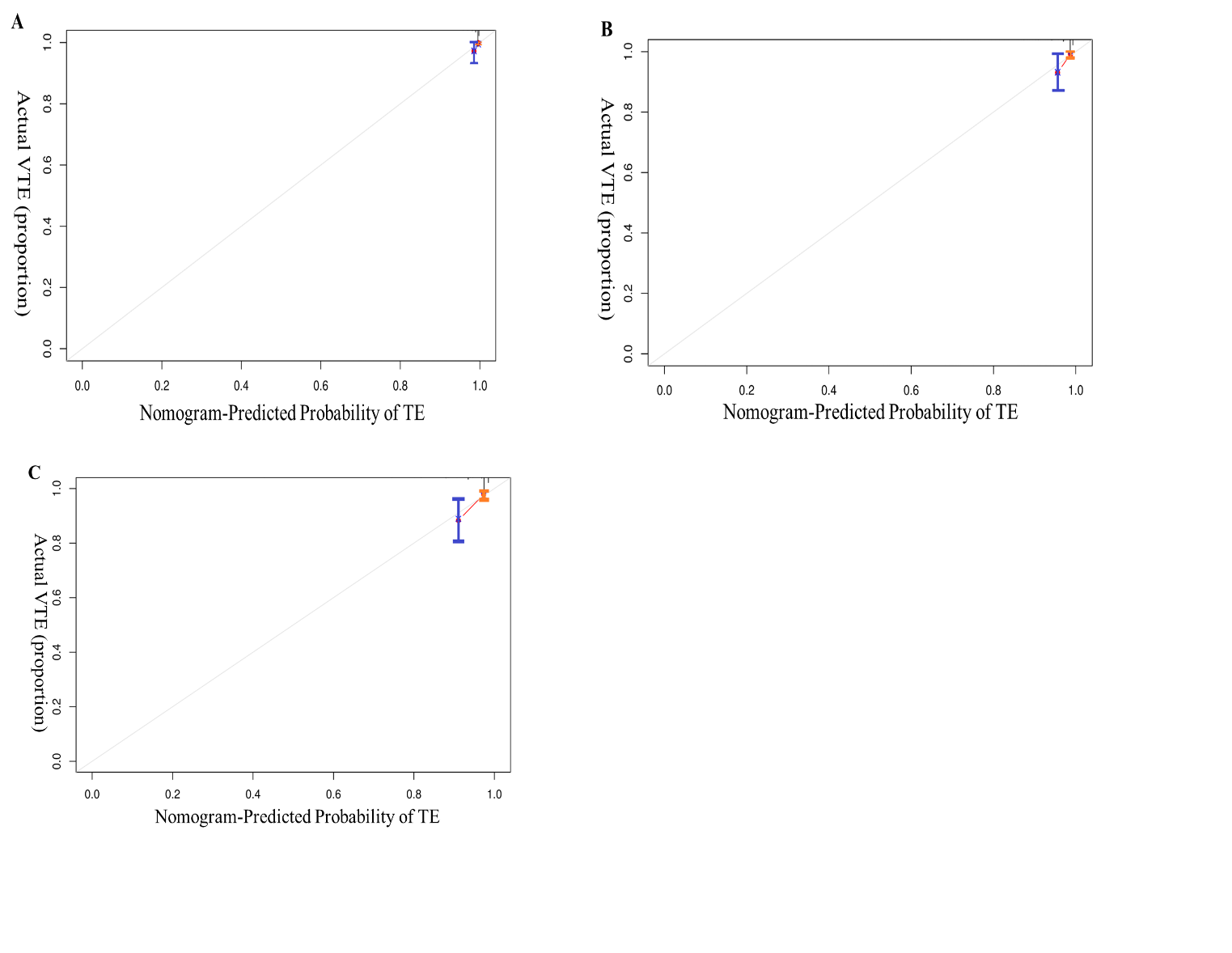


Supplement Figure 1. 0.5 years (A), 1 year (B), and 2 years(C) calibration curves for nomogram models in the model development group.


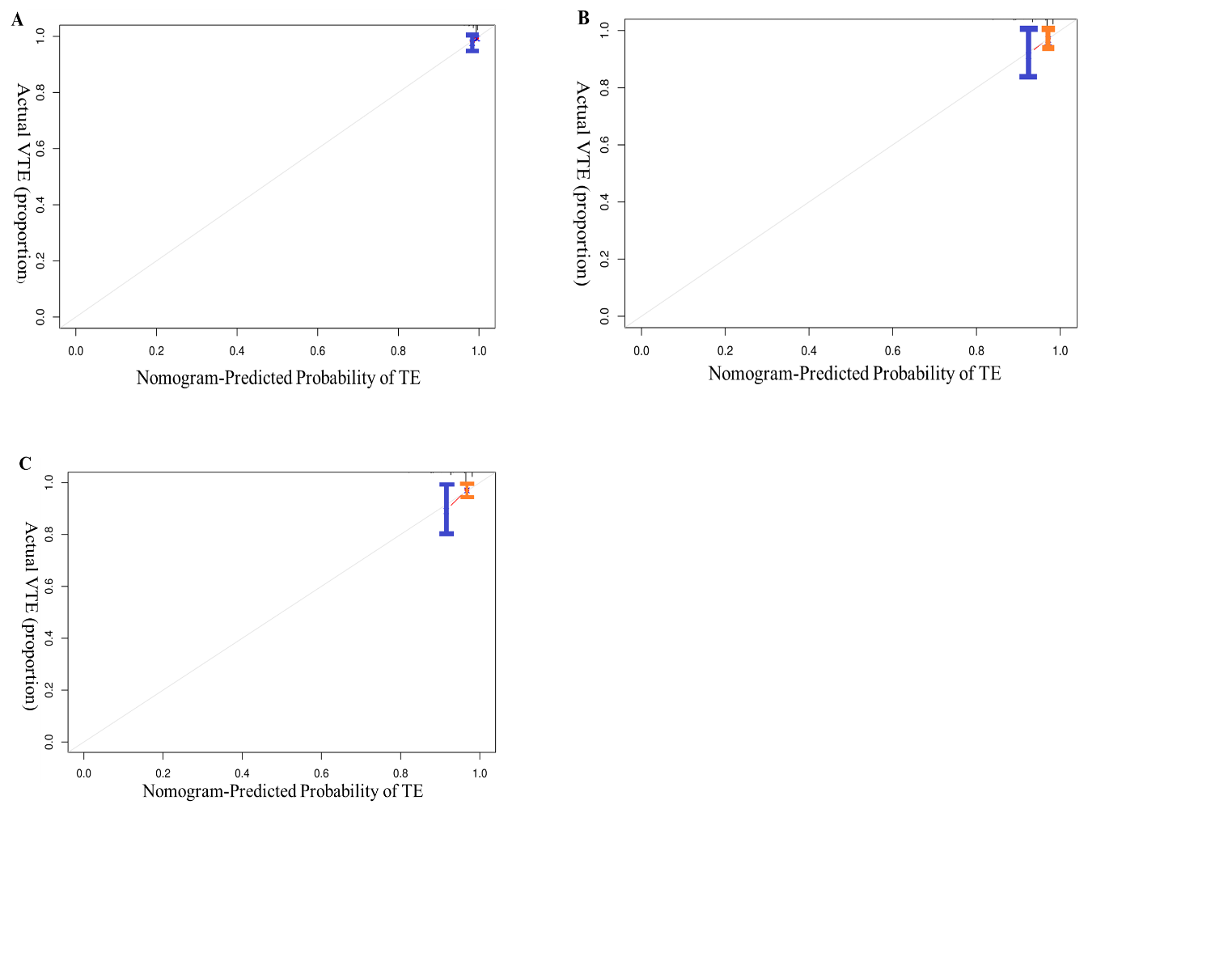


Supplement Figure 2. 0.5 years (A), 1 year (B), and 2 years(C) calibration curves for nomogram models in the validation group.
